# Integrated network pharmacology and experimental verification to reveal the mechanisms of curcumin in the treatment of colorectal cancer

**DOI:** 10.3389/fphar.2025.1703562

**Published:** 2026-01-21

**Authors:** Zhilong Yuan, Jing Hu, Canfeng Cai, Fuheng Liu, Huimou Chen, Bing Zeng

**Affiliations:** 1 Department of General Surgery, Hernia and Abdominal Wall Surgery, The Sixth Affiliated Hospital, Sun Yat-sen University, Guangzhou, China; 2 Guangdong Provincial Key Laboratory of Colorectal and Pelvic Floor Diseases, The Sixth Affiliated Hospital, Sun Yat-sen University, Guangzhou, China; 3 Biomedical Innovation Center, The Sixth Affiliated Hospital, Sun Yat-sen University, Guangzhou, China; 4 Department of Clinical Laboratory, The Sixth Affiliated Hospital, Sun Yat-sen University, Guangzhou, China; 5 Affiliated Qingyuan Hospital, The Sixth Clinical Medical School, Guangzhou Medical University, Qingyuan People’s Hospital, Qingyuan, Guangdong, China; 6 Department of Oncology, Sun Yat-sen Memorial Hospital of Sun Yat-sen University, Guangzhou, China

**Keywords:** colorectal cancer, curcumin, ferroptosis, network pharmacology, Wnt/β-catenin signaling pathway

## Abstract

**Background:**

Curcumin is a natural compound with potent anti-tumorigenic properties, demonstrating significant efficacy in treating colorectal cancer (CRC). However, the mechanisms underlying this anti-tumor effect remain unelucidated. This study aimed to explore curcumin's potential mechanisms in the CRC treatment via integrated network pharmacology, bioinformatics and experimental validation.

**Methods:**

Curcumin and CRC targets were obtained from public databases, with differentially expressed genes (DEGs) from RNA-seq. Network pharmacology-based prediction was employed to elucidate the potential mechanisms by which curcumin exerts its anti-CRC effects. Single-cell analysis was used to explore the expression of hub genes in CRC's tumor microenvironment (TME). Least absolute shrinkage and selection operator (LASSO) Cox analyses were used to construct a prognostic model. Molecular docking was employed to investigate the interactions between curcumin and hub genes. Molecular dynamics (MD) simulation was carried out to provide further verification of the findings. In vitro and xenograft mouse model experiments were conducted to validate the effects of curcumin.

**Results:**

A total of 46 potential targets were obtained. Functional enrichment analysis revealed that the potential gene set was significantly enriched in ferroptosis and the Wnt/β-catenin signaling pathway. 11 hub genes were identified from PPI network. Single-cell analysis of the hub genes indicated that their aberrant expression profiles was associated with the TME of CRC. A four-gene prognostic model, including SIRT1, SERPINE1, MMP3 and WNT5A, was constructed from the hub genes. Curcumin exerts regulatory effects on mast cells, fibroblasts, and plasma components within the context of immune modulation. Molecular docking studies showed that curcumin exhibits strong binding affinity to the hub targets with high docking scores (binding energies ≤ -6 kcal/mol), which was further confirmed by MD simulation. In addition, curcumin treatment promoted accumulation of lipid ROS (p<0.05), induced ferroptosis and activated the Wnt/β-catenin signaling pathway. Mechanistically, curcumin elicited augmented phosphorylation of GSK3β at Ser9 and reduced expression of SLC7A11 and GPX4. Furthermore, curcumin significantly inhibited tumor growth (p=0.039) and exhibited a synergistic antitumor effect with oxaliplatin in vivo.

**Conclusion:**

This study comprehensively elucidates the molecular mechanisms by which curcumin exerts its therapeutic effects in CRC via modulation of ferroptosis and Wnt/β-catenin signaling pathway. These findings provide novel mechanistic insights and support the translational potential of curcumin in preclinical and clinical frameworks.

## Graphical Abstract

**Figure:**
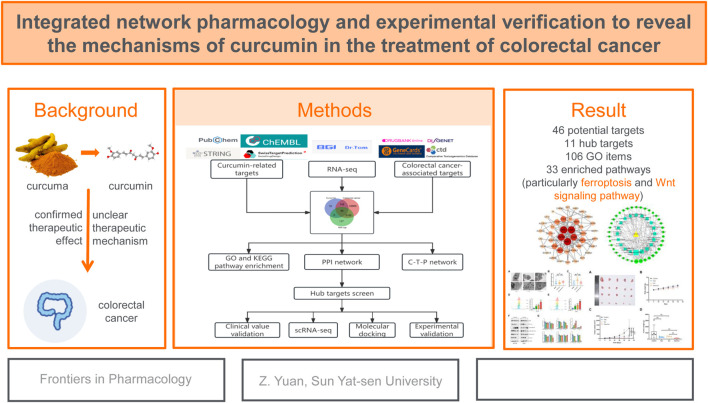

## Highlights

- Integrating network pharmacology and RNA-seq lead to more precise identification of targets of natural compound.
- Curcumin can regulate ferroptosis and WNT signaling pathway by eliciting augmented phosphorylation of GSK3β at Ser9 and reducing expression of SLC7A11 and GPX4 respectively in the treatment of colorectal cancer.
- Curcumin exert anti-tumor effect independently, but also synergistically with oxaliplatin.

## Introduction

Curcumin, chemically named diferuloylmethane, is a polyphenolic compound derived from the rhizomes of turmeric (Curcuma longa), with a chemical formula of C_21_H_20_O_6_ (Prasad et al., 2014). Accumulating evidence indicates that curcumin exerts therapeutic effects on multiple organ systems, including the nervous, cardiovascular, digestive, respiratory, endocrine, and renal systems (Ismail et al., 2019; Patel et al., 2020). Furthermore, curcumin exhibits significant pharmacological activities against multiple malignancies, particularly CRC (Shelash Al-Hawary et al., 2023).

CRC is the fourth leading cause of cancer-related mortality worldwide, accounting for nearly 900,000 deaths annually and approximately 10% of all cancer fatalities (Dekker et al., 2019). Driven by demographic aging, unhealthy dietary habits, and increasing prevalence of risk factors, the incidence and mortality of CRC continue to rise globally. Despite advances in CRC management (e.g., laparoscopic resection, cytoreductive surgery, radiotherapy, and chemotherapy), these treatment modalities have only modestly improved cure rates and long-term survival outcomes (Kuipers et al., 2015). Given the limitations of current CRC therapies, the development of novel therapeutic agents with minimal toxicity is critical for improving patient outcomes (Sung et al., 2021).

Emerging evidence suggests that novel forms of regulated cell death (RCD), including ferroptosis, are potential therapeutic targets for curcumin in CRC treatment (Fan et al., 2022). Mechanistically, curcumin exerts its anticancer effects by modulating key intracellular signaling pathways, such as the PI3K-Akt, IL-17, and cell cycle pathways, thereby inhibiting CRC cell proliferation and inducing apoptotic cell death (He et al., 2023). Recent preclinical and clinical studies have demonstrated that curcumin can modulate autophagy and apoptosis through diverse signaling networks in CRC models (Brockmueller et al., 2024). Notably, curcumin-induced ferroptosis is mediated by regulating the p53/SLC7A11/glutathione/GPX4 signaling axis, which has been independently validated (Firouzjaei et al., 2023; Ming et al., 2024). However, the underlying mechanisms of curcumin’s therapeutic effects in CRC have not been fully elucidated. Although individual studies have linked curcumin to specific signaling pathways or forms of cell death, a comprehensive understanding of its multitargeted effects in CRC—particularly the crosstalk between these pathways—remains lacking. Additionally, systematic data on the anti-CRC mechanisms of curcumin derivatives are lacking, precluding mechanistic comparisons. The multitargeted and multipathway properties of natural compounds thus highlight the need for integrative omics-based approaches to systematically characterize curcumin-related targets in CRC treatment (Zhang et al., 2023).

To fill this gap, network pharmacology has emerged as a powerful tool for deciphering the complex interactions among natural compounds, their targets, and disease pathways. Rapid advances in large-scale biomedical datasets and artificial intelligence (AI)-driven algorithms have enabled this approach to systematically characterize drug-disease interaction networks and uncover the molecular mechanisms underlying complex diseases via multi-omics integration (Crampon et al., 2022; Zhang et al., 2023; Ning et al., 2024). Previous studies have predominantly relied on public databases for target identification, which may lack sample specificity and fail to capture context-dependent molecular responses (Qu et al., 2022; Duan et al., 2025). To improve the accuracy of identifying relevant targets, this study integrated network pharmacology with RNA-seq data from curcumin-treated CRC cells. This strategy validates predicted targets using experimental data, identifies CRC-specific and curcumin-responsive molecular signatures, and prioritizes targets with both theoretical relevance and experimental evidence—thus improving the accuracy of hub gene identification.

This study aimed to identify the therapeutic targets of curcumin for CRC and elucidate the underlying molecular mechanisms using an integrated approach that combines network pharmacology and experimental validation, with the goal of providing a theoretical basis for the clinical application of curcumin in CRC treatment.

## Methods

### Cell culture

Human CRC cell lines HCT116 and DLD1 were obtained from the American Type Culture Collection (ATCC, United States). Cells were cultured in Dulbecco’s modified Eagle’s medium (DMEM; Gibco, Grand Island, NY, United States) supplemented with 10% fetal bovine serum (FBS; Gibco, Grand Island, NY, United States), 100 U/mL penicillin, and 100 μg/mL streptomycin (Gibco, United States) in a humidified incubator at 37 °C with 5% CO_2_.

### Reagents

Primary antibodies targeting GPX4, GAPDH, SLC7A11, SIRT1, SERPINE1, MMP3, phospho-GSK3β (Tyr216), phospho-GSK3β (Ser9), GSK3β, and β-catenin, as well as corresponding secondary antibodies, were purchased from Proteintech, Inc. (United States). WNT5A Rabbit mAb, as well as corresponding secondary antibodies, was purchased from Abclonal (China). Oxaliplatin (Oxa) was obtained from Selleck (United States). Curcumin and erastin were purchased from Sigma-Aldrich (United States).

### Identification of DEGs

The half-maximal inhibitory concentration (IC_50_) of curcumin in HCT116 and DLD1 cells was first determined using the Cell Counting Kit-8 (CCK-8) assay. Briefly, after cells adhered to 96-well plates, they were treated with curcumin for 48 h; CCK-8 reagent (DOJINDO, Japan) was added, and cells were cultured for an additional 4 h before measuring absorbance. To identify curcumin-responsive molecular changes in CRC cells, we performed RNA sequencing (RNA-seq) on DLD1 cells treated with curcumin or dimethyl sulfoxide (DMSO) as a control. The human CRC cell line DLD1 (ATCC, United States) was cultured as described and seeded in 96-well plates to reach 80% confluence before treatment. Cells were divided into two groups: (i) DMSO (control) and (ii) curcumin (10 μM), with 2 biological replicates per group. After 24 h of treatment, cells were cryopreserved and shipped to BGI Genomics (Shenzhen, China) for total RNA extraction, complementary DNA (cDNA) library construction, and RNA-seq using the DNBSEQ platform. We identified DEGs using DESeq2 software, with statistical significance defined as an adjusted P-value <0.05 and a log_2_ fold change (log_2_FC) ≥ 1.0.

### Identification of potential targets

We integrated DEGs from RNA-seq with curcumin- and CRC-related target libraries to identify potential disease-related gene candidates. To systematically identify potential targets mediating curcumin’s anti-CRC effects, we used a multi-step strategy. First, the SMILES (Simplified Molecular Input Line Entry System) structure of curcumin was retrieved from PubChem, and potential human targets were mined from ChEMBL using “curcumin” as a query; additional targets were supplemented via STRING database analysis and literature mining. Second, CRC-related targets were identified through a comprehensive search of GeneCards, DrugBank, DisGeNET, and Comparative Toxicogenomics Database (CTD) using the keywords “colorectal cancer”, “colon cancer”, and “rectal cancer”. To ensure high relevance to CRC, the “score” threshold was set to the median value, and genes with a score above the median were selected to establish a CRC-related target library. Duplicate entries were removed, and target names were standardized using UniProt. Finally, DEGs from RNA-seq were integrated with the curcumin and CRC target libraries to generate potential disease-gene candidates.

### Enrichment analysis

To gain insights into the biological functions and signaling pathways associated with the 46 potential targets, Gene Ontology (GO) and Kyoto Encyclopedia of Genes and Genomes (KEGG) enrichment analyses were conducted using the Database for Annotation, Visualization and Integrated Discovery (DAVID) database. The analysis was restricted to “*Homo sapiens*” gene annotations, with significance set at P < 0.05 and False Discovery Rate (FDR) < 0.05. The top 10 terms for Biological Process (BP), Cellular Component (CC), and Molecular Function (MF), as well as the top 20 KEGG signaling pathways, were imported into bioinformatics software for visualization and further analysis.

### Molecular mechanism analysis

PPI and drug-target-pathway (DTP) networks were constructed using the STRING database (version 11.5) with a minimum confidence score of 0.5 and species filter set to *Homo sapiens*. The resulting network files were imported into Cytoscape 3.9.1 for topological analysis and visualization. Hub targets were identified using the CytoHubba plugin’s degree centrality algorithm, which prioritizes nodes based on four topological parameters: (i) betweenness centrality > median, (ii) closeness centrality > median, (iii) average shortest path length > median, and (iv) degree value > median (Huang, 2023). These parameters filtered nodes with significant topological roles in the PPI network. Additionally, we used the MCODE plugin to validate module enrichment of the identified hub targets, confirming their biological relevance within functional pathways.

### Immune correlation analysis

Given the critical role of the tumor microenvironment (TME) in CRC progression and therapy response, we next analyzed the correlation between hub gene expression and immune cell infiltration in CRC. The spatial expression patterns and cluster-specific distribution of hub genes were systematically evaluated in the CRC_GSE146771_Smart-seq2 single-cell RNA-seq dataset using the Tumor Immune Single-cell Hub (TISCH) database (http://tisch.comp-genomics.org/). This analysis examined 22 distinct immune cell populations (including macrophages, T lymphocytes, and dendritic cells) to characterize their infiltration patterns. The ggstatsplot and ggplot2 R packages were used to perform nonparametric correlation analysis between hub gene expression levels and immune cell infiltration scores, while associations with immune checkpoint molecules were quantified using partial correlation controlling for batch effects. We performed decision curve analysis (DCA) for different models using the ggDCA package in R software.

### Establishment of the prognostic model

To evaluate the clinical relevance of the identified hub genes, a CRC dataset containing 620 samples was downloaded from The Cancer Genome Atlas (TCGA) database for gene expression and prognostic analysis. Statistical analysis was performed using R software (version 4.4.3), with significance defined as P < 0.05. To identify the most critical prognostic genes, a penalized Cox regression approach with the least absolute shrinkage and selection operator (LASSO) penalty was applied using Assistant for Clinical Bioinformatics (http://www.aclbi.com/) to establish a prognostic model. The CIBERSORT algorithm was used to determine the relative proportions of 22 infiltrating immune cell types, and immunological scores for individual samples were calculated using the “ESTIMATE” algorithm.

### Molecular docking

To validate the direct interactions between curcumin and the identified hub targets, molecular docking was performed. The RCSB Protein Data Bank (PDB) database (https://www.rcsb.org/) was used to retrieve protein structures of candidate targets, and PyMOL software was employed for pre-docking processing (e.g., removing ligands and non-protein molecules). The three-dimensional (3D) structure of curcumin was optimized using ChemDraw software and imported into AutoDock Tools 1.5.7 for hydrogenation, charge assignment, and calculation of rotatable bonds. Semi-flexible molecular docking was performed using AutoDock Tools 1.5.7, and the clustering tool was used to select the lowest-energy conformation as the research object. Docking results were visualized using PyMOL software, with a binding free energy ≤ −6 kcal/mol considered indicative of strong binding affinity.

### Molecular dynamics simulation

To confirm the stability of the predicted curcumin-target interactions under physiological conditions, we conducted MD simulations on two key hub targets, given their central role in the key pathway. Simulations were performed using GROMACS 2022 software: small molecules were modeled using the General Amber Force Field (GAFF), and proteins were described using the AMBER14SB force field combined with the TIP3P water model to construct the complex simulation system. Simulations were run under constant temperature (298 K) and pressure (1 bar) conditions with periodic boundary conditions. All hydrogen bonds were constrained using the LINCS algorithm with an integration step of 2 fs; electrostatic interactions were calculated using the particle mesh Ewald (PME) method, and the cutoff value for non-bonded interactions was set to 10 Å with updates every 10 steps. After 100 ps of NVT (constant number of particles, volume, and temperature) and NPT (constant number of particles, pressure, and temperature) equilibrium simulations, 100 ns of MD simulations were performed for the complex system, with conformations saved every 10 ps. Simulation trajectories were analyzed using VMD and PyMOL software.

### Transmission electron microscopy

For transmission electron microscopy (TEM) analysis, HCT116 cells were cultured in 96-well plates and treated with DMSO, curcumin (20 μM), or curcumin (20 μM) combined with Ferrostatin-1 (Fer-1) (5 μM) for 24 h. Cells were fixed with 2.5% glutaraldehyde in PBS for 24 h, washed with 0.1 M PBS, and treated with 0.1% Millipore-filtered cacodylate-buffered tannic acid. After post-fixation with 1% buffered osmium tetroxide and staining with 1% Millipore-filtered uranyl acetate, samples were dehydrated, embedded, and incubated in a 60 °C oven for 24 h. Digital images were obtained using a transmission electron microscope, and mitochondrial density was quantitatively analyzed using ImageJ software.

### Lipid ROS assay

HCT116 cells and DLD1 cells were cultured in 96-well plates and treated with DMSO, curcumin (5 μM), curcumin (10 μM), curcumin (20 μM), or curcumin (20 μM) combined with Fer-1 (5 μM) for 24 h respectively. Lipid reactive oxygen species (ROS) levels were detected using the C11-BODIPY 581/591 kit (Thermo Fisher, United States) strictly according to the manufacturer’s protocol. These cells were treated as described, thoroughly washed with PBS, and incubated with 2 μM C11-BODIPY 581/591 in a humidified incubator for 30 min. After removing unbound probes via washing, cells were detached with trypsin, collected, and analyzed by flow cytometry to assess fluorescence intensity as an indicator of lipid ROS levels.

### Western blot analysis

Cell lysates were collected using lysis buffer supplemented with protease and phosphatase inhibitors and kept on ice for 30 min. Lysates were centrifuged at 12,000 rpm for 15 min at 4 °C, and supernatants were collected for protein quantification using the Bradford assay. Protein samples were loaded onto sodium dodecyl sulfate-polyacrylamide gel electrophoresis (SDS-PAGE) gels, electrophoresed, and transferred to polyvinylidene fluoride (PVDF) membranes. Membranes were blocked with 5% non-fat milk, washed with TBST, and incubated with corresponding primary antibodies overnight at 4 °C. After incubation with horseradish peroxidase (HRP)-conjugated secondary antibodies for 1 h at room temperature, immunoreactive bands were detected using an ECL system and an image reader. Densitometric analysis was performed using ImageJ, with data corrected by background subtraction and normalized to GAPDH as an internal control.

### Xenograft experiment

To translate the *in vitro* findings to an *in vivo* setting, CRC xenograft models were established in nude mice. Four-week-old male immunodeficient BALB/c nu/nu mice were obtained from BesTest Biotechnology (China) and housed under specific pathogen-free (SPF) conditions at 22 °C–24 °C with a 12-h light/dark cycle for 3 days of acclimation. DLD1 cells (1 × 10^6^ cells in 0.1 mL serum-free medium) were subcutaneously injected into the right flanks of nude mice. Once tumors reached an approximate volume of 50 mm^3^, mice were randomized into four experimental groups (n = 5/group): (i) Control (0.9% saline), (ii) Oxa (oxaliplatin, 10 mg/kg), (iii) Cur (curcumin, 50 mg/kg), and (iv) Oxa + Cur (oxaliplatin 10 mg/kg + curcumin 50 mg/kg). Treatments were administered intraperitoneally twice a week. On day 30, mice were euthanized via cervical dislocation, tumors were surgically excised, and tumor weights and volumes were recorded. Slices (5 μm) of paraffin-embedded tumor tissues were incubated with SLC7A11 or GPX4 antibody at 4 °C for one night. Next, horseradish peroxidase (HRP)-conjugated secondary antibody was applied to these slices for 30 min of incubation at room temperature. Fresh 3,3'-diaminobiphenylamine solution was added to the slices, and they were counterstained with hematoxylin. Pictures were photographed with a microscope. All animal procedures were approved by the Institutional Animal Care and Use Committee (IACUC) of Sun Yat-sen University.

### Statistical analysis

Statistical analyses were conducted using SPSS (Version 26.0) and R (Version 4.4.3) software. Categorical variables were compared between groups using the χ^2^ test or Fisher’s exact test. Continuous variables were analyzed via independent samples t-test or Mann–Whitney U test based on data distribution. All statistical tests were two-tailed, with significance defined as p < 0.05.

## Results

### Identification of potential targets

**FIGURE 1:**
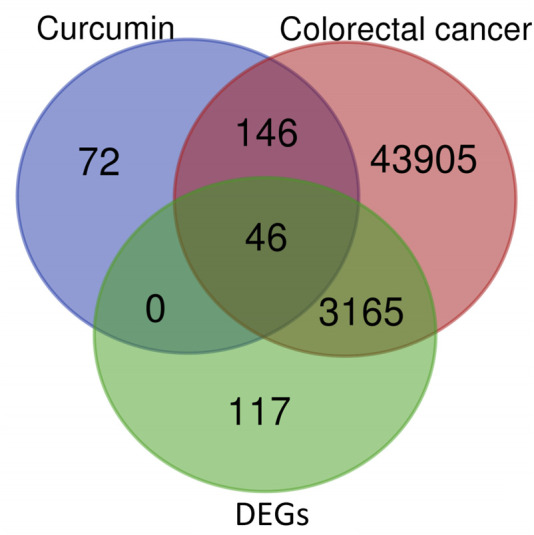
Venn diagram of curcumin targets, CRC-related genes, and DEGs from curcumin-treated DLD1 cells.

The IC_50_ of curcumin in HCT116 and DLD1 cells was showed in Supplementary Figure S1. A total of 264 targets of curcumin and 47,261 CRC-related targets were initially screened from public databases. To address the imprecision of target identification based solely on databases, we combined these targets with RNA-seq data from curcumin-treated DLD1 cells: four samples yielded an average of 6.68 Gb of data per sample, with an average alignment rate of 92.06% to the human genome and 70.64% to the gene set, resulting in the detection of 17,604 genes and identification of 3,328 DEGs. Intersection analysis using Venny 2.1.0 narrowed these down to 46 overlapping targets as potential candidates (Figure 1), which are listed in Supplementary Table S1. To further explore the biological relevance of these 46 targets, we next performed functional enrichment analyses.

### GO and KEGG pathway enrichment analysis

**FIGURE 2:**
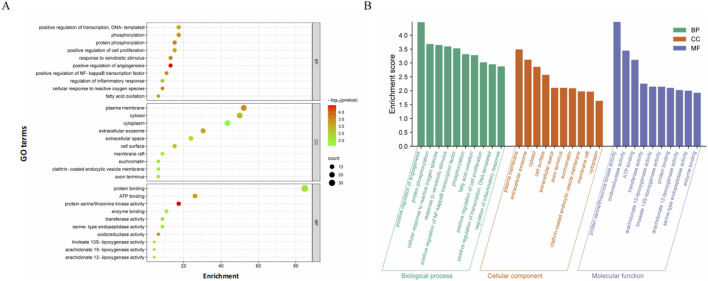
GO enrichment analysis of potential targets (top 10). **(A)** These enriched entries highlighted key biological processes, cellular components, and molecular functions that are potentially influenced by curcumin. **(B)** This histogram illustrated the enrichment score of top 10 enriched entries for each GO category (BP, CC, and MF).

**FIGURE 3:**
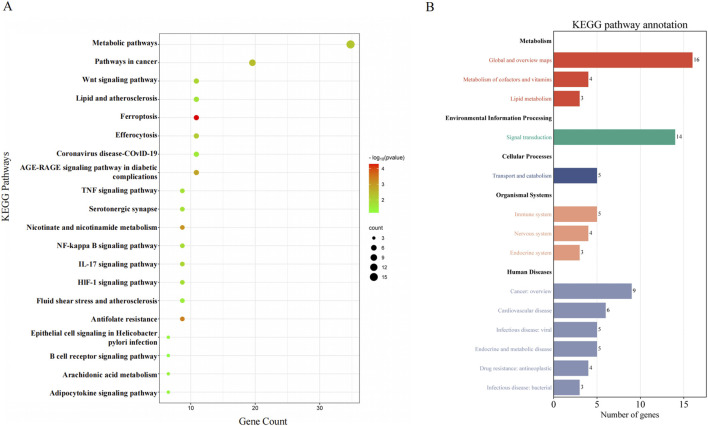
KEGG enrichment analysis of potential targets (top 20). **(A)** The bubble diagram visualized the top 20 enriched KEGG signal pathways in reverse order of FDR values. **(B)** The histogram illustrated the frequency and significance of enrichment for each pathway.

GO and KEGG enrichment analyses using the DAVID database (restricted to *Homo sapiens*) identified 106 statistically significant GO terms, including 70 biological processes (BP), 11 cellular components (CC), and 25 molecular functions (MF). GO terms were ranked by FDR value, and the top 10 terms for BP, CC, and MF were visualized (Figures 2A,B). Integrated DAVID and FUMA GWAS analysis identified 33 significantly enriched pathways (FDR<0.05), with the top 20 KEGG pathways visualized via bubble plots and classification histograms (sorted by FDR) (Figure 3A). Notably, these genes are widely distributed across various subcellular localizations and are involved in key regulatory processes such as the cell cycle, cell proliferation, and cell signaling. The top enriched KEGG pathways mainly included ferroptosis, the Wnt/β-catenin signaling pathway, nicotinate and nicotinamide metabolism, and efferocytosis—pathways primarily involved in metabolism, human diseases, and environmental information processing (Figure 3B).

### Establishment of PPI network and DTP network

**FIGURE 4:**
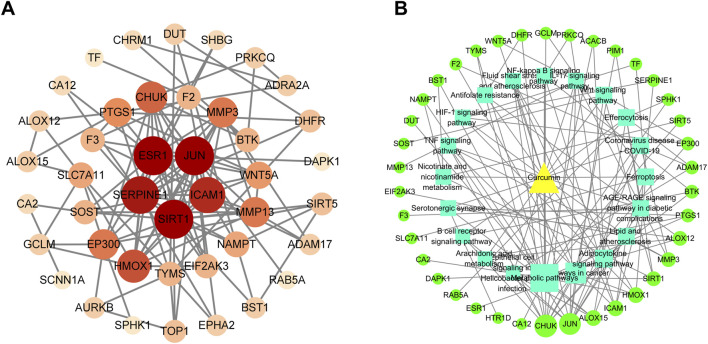
Network graph **(A)** PPI network of potential targets for curcumin therapy was constructed based on 46 potential targets. **(B)** Drug-pathway-target network was constructed based on 20 KEGG enrichment pathways and 46 potential targets.

The STRING database (version 11.5) was used to analyze the 46 candidate targets, generating a PPI network with 42 nodes and 232 edges (average node degree = 5.52) (Figure 4A). Topological analysis using CytoHubba identified 11 hub targets based on the four aforementioned criteria: ESR1, JUN, SIRT1, SERPINE1, ICAM1, HMOX1, CHUK, EP300, MMP3, PTGS1, and WNT5A (Table 1). Utilization of the MCODE plugin revealed that the top module (score = 8.364) included 10 of the 11 hub genes (ESR1, JUN, SIRT1, SERPINE1, ICAM1, HMOX1, CHUK, MMP3, and WNT5A) and was significantly enriched in the Wnt/β-catenin signaling pathway (FDR = 2.4 × 10^−2^), validating the biological relevance of the identified hub targets (Supplementary Figure S2). These hub targets and KEGG pathways were categorized and organized to construct the DTP network in Cytoscape 3.9.1 (Figure 4B), where the yellow triangle represents curcumin, green circles represent potential targets, and cyan squares represent enriched pathways.

**TABLE 1:** Hub targets screened from PPI network.

Gene	Degree	Closeness	Betweenness	Clustering coefficient
ESR1	15	27.3	308.85286	0.32381
JUN	15	26.8	186.83905	0.38095
SIRT1	15	27.0	238.25158	0.38095
SERPINE1	14	25.8	194.89686	0.31868
ICAM1	13	25.4	126.9618	0.46154
HMOX1	11	24.4	97.69396	0.52727
CHUK	10	24.1	179.93196	0.42222
EP300	9	23.3	193.94487	0.22222
MMP3	9	22.8	20.55675	0.72222
PTGS1	8	22.4	213.24445	0.10714
WNT5A	7	22.0	46.29547	0.57143

### Immune landscape and hub gene correlations

**FIGURE 5:**
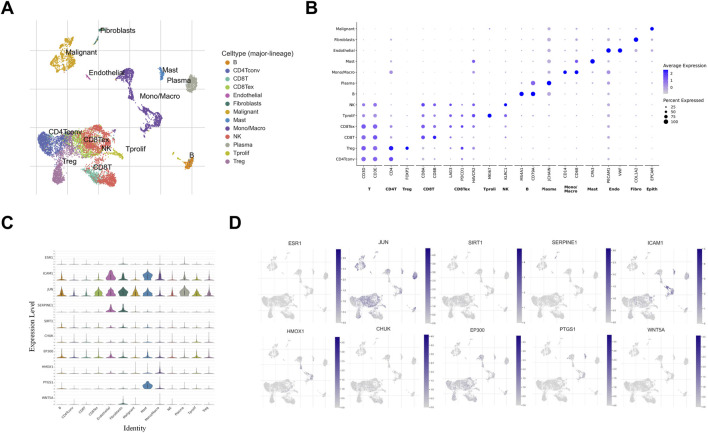
Immune landscape and hub gene correlations **(A)** Seurat clustering of all immune cells in CRC. **(B)** The bubble plot of the expression between immune cell subgroups and their marker gene expression. **(C)** The violin - plot from single - cell sequencing data, depicting the expression levels of hub genes, there is a close and significant association between the subsets of immune cells and hub targets. **(D)** UMAP feature plots depicting single-cell gene expression of hub genes.

Comprehensive correlation analysis was performed to characterize the relationship between hub gene expression and the TME in CRC, with the workflow for processing the GSE146771 Smart-seq2 dataset visualized in Figure 5A. As shown in Figure 5B, the average expression level and percent expressed of various markers across different cell types are illustrated. Figures 5C,D shows distribution of expression levels of core target genes in different cell subsets and the expression level distribution of hub target genes across distinct cell subsets, and the UMAP-based expression distribution of these hub target genes in the single-cell population, with JUN (36.5% ± 33.0%), EP300 (19.5% ± 27.7%), and ICAM1 (18.1% ± 24.4%) emerging as the top genes with the highest expression proportions among hub genes (Supplementary Table S2). Supplementary Figure S3 shows the correlation between hub genes and immune cell infiltration. PTGS1 exhibited a significant positive correlation with mast (r = 0.495, p < 0.001) and mono/macro (r = 0.310, p < 0.001). What’s more, HMOX1 was positively correlated with mono/macro (r = 0.314, p < 0.001). As an example, PTGS1 high-expression cohorts showed significantly greater mast cell infiltration than low-expression cohorts (4.62 ± 4.74 vs. 0.26 ± 0.35, p < 0.05), thereby confirming functional links between hub genes and TME composition.

### Prognosis value of hub genes in CRC

**FIGURE 6:**
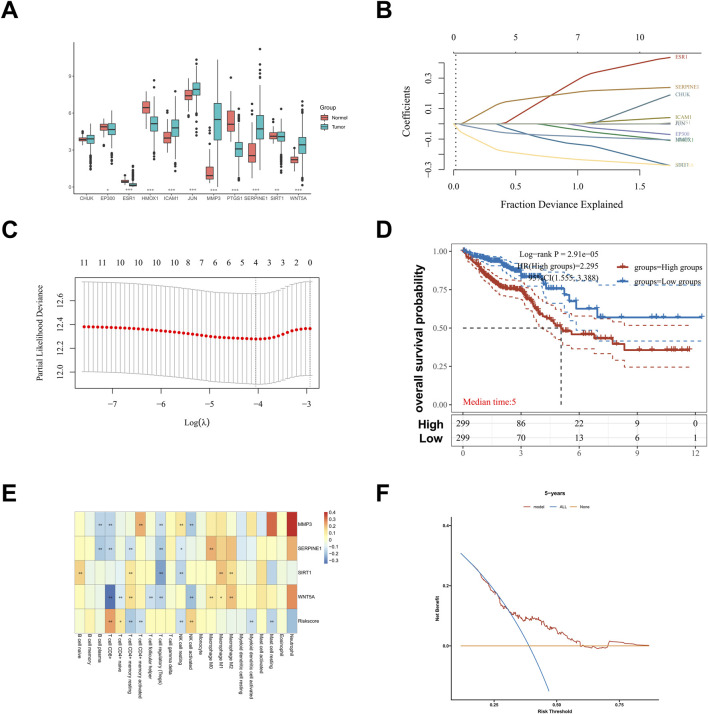
Establishment and verification of the prognostic model. **(A)** The expression plot derived from TCGA sequencing of CRC, visualizing the expression of hub genes. **(B)** LASSO coefficient spectra of 11 hub genes were included, and coefficient distribution maps of log (λ) sequences were generated. **(C)** The selection of optimal ferroptosis-related prognostic model (FPM) parameters in LASSO model (λ). **(D)** Survival status of the patients. More dead patients corresponding to the higher risk score. **(E)** Heatmap of the expression profiles of the four prognostic genes in low-risk and high-risk group. **(F)** The 5-years survival curve for further validation of the established prognostic model, ensuring its reliability and generalizability.

To develop a CRC-associated prognostic model, univariate Cox regression analysis was first performed on the 11 hub genes and clinical data from CRC patients. Differential expression analysis revealed significantly lower mRNA levels of ESR1, HMOX1, and SIRT1 (P < 0.05), as well as elevated PTGS1 expression (P < 0.05), in CRC tissues compared to normal controls (Figure 6A). Subsequently, LASSO Cox regression analysis was performed on these 11 candidate genes to optimize feature selection (Figures 6B,C). Through comprehensive regression analysis, four genes (SIRT1, SERPINE1, MMP3, and WNT5A) with significant prognostic value (P < 0.05) were identified for inclusion in the predictive model. The risk score was calculated using the formula: risk score = −0.010 × Expr (SIRT1) + 0.134 × Expr (SERPINE1) - 0.0548 × Expr (MMP3) - 0.1754 × Expr (WNT5A). Patients were stratified into high-risk and low-risk cohorts based on individual risk scores derived from mRNA expression profiles, and Kaplan-Meier analysis demonstrated significant survival differences between these groups (Figure 6D). CIBERSORT-based immune infiltration analysis revealed significant negative correlations between risk scores and CD8^+^ T cells, resting mast cells, and activated dendritic cells (Figure 6E). “None” and “ALL” represent two reference lines (the closer the curves of other models are to these lines, the lower their clinical utility), and DCA indicated that the four-gene prognostic model could predict CRC outcomes better than either the “all-hub targets” or “no-hub target” schemes (Figure 6F). The other validation data of our prognostic model are supplemented in Supplementary Figure S4.

### The interaction between curcumin and targets

**FIGURE 7:**
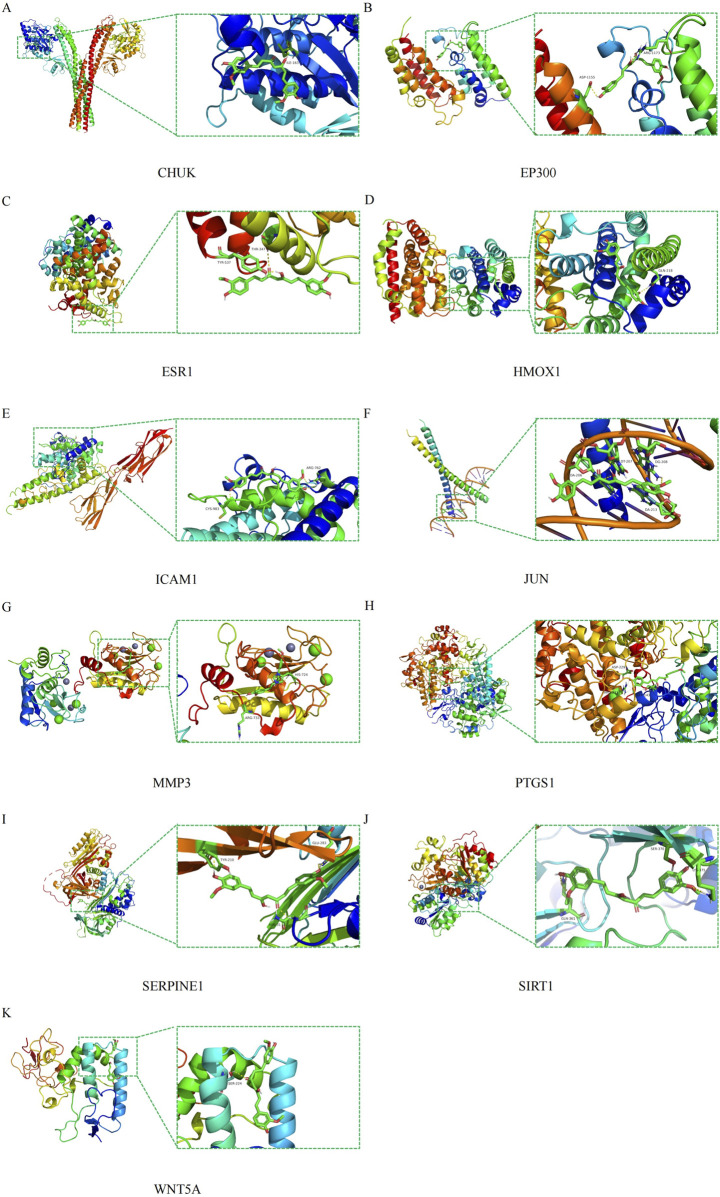
Molecular docking pattern of curcumin and core target protein. **(A)** CHUK, **(B)** EP300, **(C)** ESR1, **(D)** HMOX1, **(E)** ICAM1, **(F)** JUN, **(G)** MMP3, **(H)** PTGS1, **(I)** SERPINE1, **(J)** SIRT1, **(K)** WNT5A.

To validate the direct interaction between curcumin and the identified hub targets, molecular docking and MD simulations were performed. Based on PPI network analysis, 11 hub targets were selected for molecular docking studies with curcumin. Structural data of these targets (ESR1, JUN, SIRT1, SERPINE1, ICAM1, HMOX1, CHUK, EP300, MMP3, PTGS1, and WNT5A) were retrieved from the Protein Data Bank (PDB). The three-dimensional structure of curcumin was optimized and processed using AutoDock tools. Systematic molecular docking simulations revealed specific binding patterns between curcumin and all investigated targets, with detailed interaction types (hydrogen bonds, hydrophobic interactions) and bond lengths documented in Table 2. Visualization of three-dimensional binding complexes demonstrated stable interactions between curcumin and key amino acid residues across all targets (Figures 7A–K). All targets showed strong binding affinity (binding energies < −6 kcal/mol, range: 6.8 to −9.5 kcal/mol). This robust computational evidence suggests curcumin’s multi-target potential through direct interactions with CRC-related hub proteins, possibly contributing to its therapeutic effects against CRC.

**TABLE 2:** Molecular docking.

Gene	Entry ID	Link site	Affinity (kcal/mol)
ESR1	1L2I	THR-347, TYR-537	−6.3
JUN	2H7H	DA-206, DT-207, DG-208, DA-213	−7.9
SIRT1	8BBK	GLN362, SER-370, LYS-375	−8.1
SERPINE1	7AQF	ASN-31, TYR-210, GLU-283	−7.4
ICAM1	5MZA	ARG-762, CYS-983	−6.8
HMOX1	3CZY	GLN-218	−6.8
CHUK	5TQY	ILE-163	−7.0
EP300	8FVS	ASP1155, ARG-1173	−8.7
MMP3	1D7X	HIS-724, ARG-733	−9.5
PTGS1	3N8Y	SER-143, ARG-229, ARG-333	−8.3
WNT5A	9FSE	SER-224	−6.5

### Molecular dynamics simulation

**FIGURE 8:**
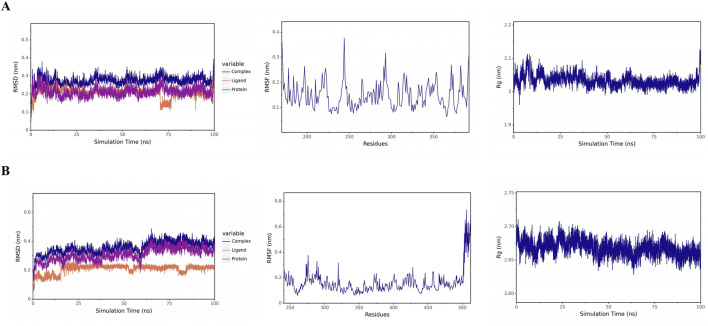
Molecular dynamics simulation analysis. **(A)** RMSD, RMSF, Rg analysis of the WNT5A-curcumin complex. **(B)** RMSD, RMSF, Rg analysis of the SIRT1-curcumin complex.

Root mean square deviation (RMSD) was used to assess the stability of the simulated systems, with an RMSD value within 1 nm indicating relative stability of protein-ligand interactions in a physiological environment. Under physiological conditions, the WNT5A-curcumin complex stabilized at 0.27 nm after 5 ns, with the radius of gyration (Rg) changing from 2.01 to 2.08 nm; the SIRT1-curcumin complex stabilized at 0.39 nm at 10 ns, with Rg ranging from 2.70 to 2.64 nm (Figures 8A,B). Both complexes showed reduced residue fluctuations via root mean square fluctuation (RMSF), confirming stable binding interfaces. These MD metrics validated long-term complex stability and provided dynamic evidence for docking-predicted interactions, bridging static docking results with physiological relevance. Building on these *in silico* findings, we next validated curcumin’s effects on ferroptosis and the Wnt/β-catenin pathway through *in vitro* experiments.

### *Curcumin promotes ferroptosis and regulates Wnt*/β-catenin *signaling pathway in vitro*

**FIGURE 9:**
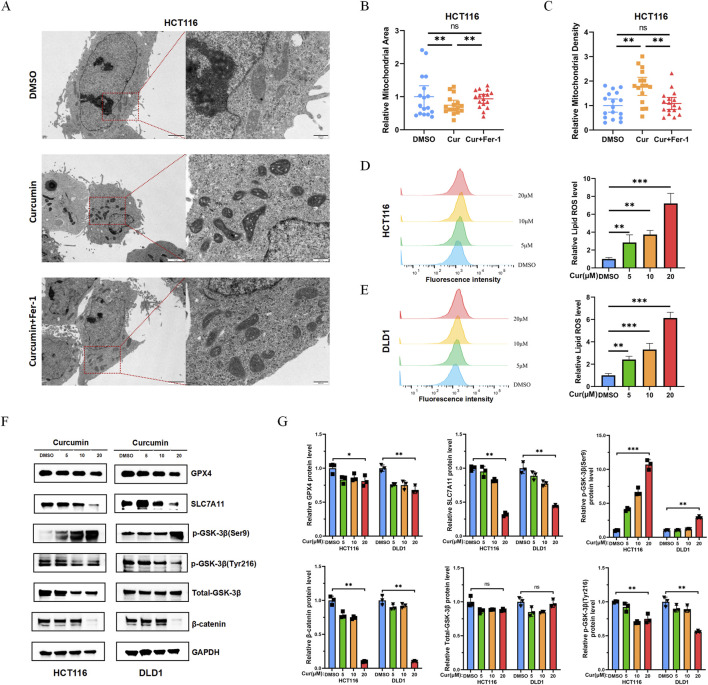
Curcumin promotes ferroptosis and regulates Wnt/β-catenin signaling pathway *in vitro*. **(A)** Representative images of transmission electron microscopy observation of mitochondria; curcumin induced reduced mitochondrial volume and increased membrane density in HCT116 cell lines (scale bar: 2 μm and 500 nm); **(B)** Relative mitochondrial area in HCT116 cell lines. **(C)** Relative mitochondrial density in HCT116 cell lines. **(D–E)** Curcumin induced the accumulation of Lipid ROS in CRC cells. Dose-dependent accumulation of Lipid ROS in HCT116 and DLD1 cells treated with curcumin (5–20 μm). **(F–G)** Curcumin effectively inhibited the expression of SLC7A11 and GPX4, downregulated level of β-catenin and promoted phosphorylation of GSK3β at Ser9. *P < 0.05, **P < 0.01 and ***P < 0.001.

Ferroptosis is characterized by a decrease in mitochondrial volume and an increase in membrane density (Stockwell et al., 2020). TEM revealed that curcumin treatment induced these characteristic ferroptosis-related alterations in CRC cells, including mitochondrial shrinkage and increased membrane density in HCT116 cells (Figure 9A). The Fer-1 significantly attenuated these morphological changes in both HCT116 (p = 0.015) and DLD1 cells (p = 3.8 × 10^−10^) (Figures 9B,C). Dose-dependent lipid peroxidation was observed with curcumin treatment (5–20 μM), peaking at 20 μM with a 3.1-fold increase in lipid ROS compared to untreated controls (P < 0.001, Figures 9D,E). Curcumin treatment significantly promotes the release of lipid ROS in CRC cells, and this effect is remarkably reversed by the addition of Fer-1, indicating that curcumin-induced lipid ROS release is mainly mediated through ferroptosis (Supplementary Figures S5,S6). To identify the specific molecular mechanism of curcumin in regulating ferroptosis, the effects of different curcumin concentrations on SLC7A11 and GPX4 expression were detected: mechanistically, curcumin downregulated these key ferroptosis regulators in CRC cells (Figures 9F,G). Concomitant modulation of the Wnt/β-catenin pathway was evidenced by enhanced GSK3β phosphorylation at Ser9 and reduced β-catenin levels, suggesting dual regulation of ferroptosis and oncogenic signaling. Curcumin treatment significantly downregulates the expression levels of SIRT1, SERPINE1, MMP3, and WNT5A, indicating that they may be direct targets of curcumin in CRC (Supplementary Figure S7).

### Curcumin inhibited tumor growth *in vivo*

**FIGURE 10:**
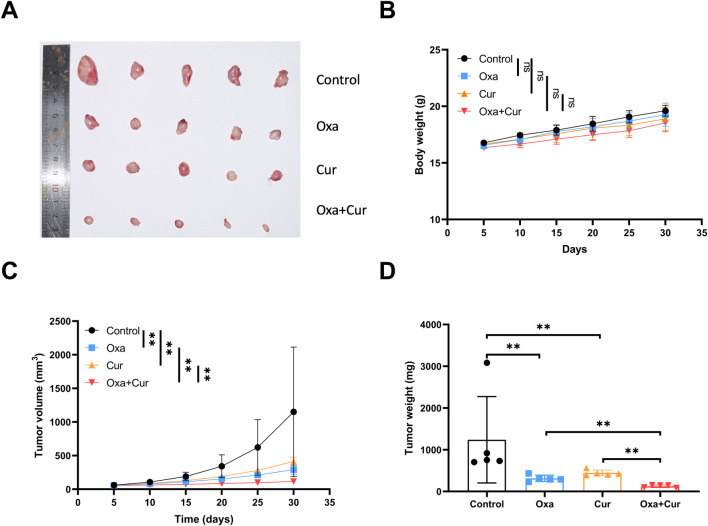
Curcumin inhibited tumor growth *in vivo* **(A)** The representative images of burdened tumors in each group. **(B)** The treatment had no significant effect on the body weight of mice. **(C–D)** Curcumin and oxaliplatin significantly suppressed the volume and weight of xenograft. *P < 0.05, **P < 0.01 and ***P < 0.001.

Body weight monitoring showed no significant treatment-related toxicity, (Figures 10A,B), indicating good tolerability of curcumin and its combination with oxaliplatin. Tumor volumes were 1,152 ± 963, 381 ± 100, 325 ± 79, and 115 ± 29 mm^3^ for the control, oxaliplatin, curcumin, and oxaliplatin + curcumin groups, respectively (Figures 10C,D). The results demonstrated that both curcumin (p = 0.039) and oxaliplatin (p = 0.053) significantly suppressed CRC proliferation in nude mice. Furthermore, combination therapy with curcumin and oxaliplatin exhibited a more pronounced inhibitory effect on tumor proliferation compared to monotherapy with either agent alone (p = 0.034), suggesting potential synergistic antitumor activity. Moreover, the immunohistochemical results demonstrated that curcumin could significantly reduce the expression levels of SLC7A11 and GPX4 in tumor tissues (Supplementary Figure S8).

## Discussion

Curcumin, a natural polyphenol derived from Curcuma longa, is a promising candidate for CRC therapy, exhibiting antitumor efficacy by modulating invasion/metastasis pathways and associated molecular mechanisms (Zhang et al., 2016; Ismail et al., 2019). Despite encouraging preclinical and clinical evidence, the precise molecular pathways underlying curcumin’s therapeutic effects in CRC remain incompletely defined. As an integrated analytical tool, network pharmacology provides an innovative framework for deciphering evidence-based associations, enabling the characterization of complex crosstalk among diseases, biological systems, and natural compounds through the ‘curcumin-protein/gene-colorectal cancer’ paradigm. (Li and Zhang, 2013; Wang et al., 2021). Notably, our study differs from previous work by integrating network pharmacology with experimental validation, particularly emphasizing the use of RNA sequencing data from curcumin-treated CRC cells. This approach addresses a critical limitation of previous network pharmacology studies, which predominantly relied on public databases for target prediction, thus potentially overlooking context-specific, curcumin-responsive molecular signatures in CRC. By contrast, our strategy prioritizes targets with both theoretical relevance derived from network pharmacology analysis and experimental evidence of differential expression, as determined through RNA-seq. This integrated approach thus improves the precision of hub gene identification.

In our analysis, 46 overlapping targets were recognized as potential candidates following the integration of database-derived targets and RNA-seq data. Among these, pathway enrichment analysis highlighted ferroptosis and the Wnt/β-catenin signaling pathway as two central axes of curcumin’s action, which aligns with emerging evidence linking these pathways to CRC progression (Saini et al., 2023; Yen et al., 2019). Eleven hub genes (ESR1, JUN, SIRT1, SERPINE1, ICAM1, HMOX1, CHUK, EP300, MMP3, PTGS1, and WNT5A) were prioritized based on topological properties within the PPI network, and these genes are known to orchestrate invasive phenotypes and poor prognosis through pathways such as ferroptosis, extracellular matrix remodeling, inflammation-oxidative stress cycles, and metabolic adaptation. Immune correlation analysis using the TISCH database further confirmed a close and significant association between hub targets and the immune microenvironment of CRC patients, underscoring the potential of these genes to modulate TME composition—a key determinant of therapy response in CRC.

To evaluate the clinical relevance of these hub genes, we developed a four-gene prognostic model (SIRT1, SERPINE1, MMP3, WNT5A) using LASSO Cox regression analysis. Kaplan-Meier analysis demonstrated significant survival differences between high-risk and low-risk cohorts, indicating that these genes are robust prognostic markers for CRC. This model also outperformed both “all-hub targets” and “no-hub target” schemes in DCA, highlighting its potential clinical utility for risk stratification and personalized therapy planning. Molecular docking and MD simulations further validated the direct interaction between curcumin and the identified hub targets, with stable binding observed for all 11 hub genes (binding energies < -6 kcal/mol) and long-term stability confirmed for WNT5A and SIRT1—key regulators of the Wnt/β-catenin signaling pathway.

In our study, network pharmacology analysis identified ferroptosis as a key pathway in curcumin’s therapeutic action. Ferroptosis, an iron-dependent form of regulated cell death (RCD) characterized by lipid peroxidation, has emerged as a promising therapeutic target in CRC (Saini et al., 2023). Experimental validation showed that curcumin promoted accumulation of lipid ROS in CRC cells, accompanied by distinctive mitochondrial morphological changes, which were reversed by ferroptosis inhibitors. Mechanistically, curcumin effectively suppressed expression of SLC7A11 and GPX4, key regulators of ferroptosis. These findings align with preclinical studies demonstrating curcumin-induced ferroptosis in osteosarcoma cells via downregulation of SLC7A11 and GPX4, and its role in CRC through modulation of the p53/SLC7A11/glutathione/GPX4 axis (Lin et al., 2021; Ming et al., 2024). Notable discrepancies exist in the literature, with some studies reporting curcumin-mediated upregulation of GPX4 via Nrf2/ARE pathway activation in cardiac myocytes and chondrocytes, likely attributable to cell-type specific responses (Zhou et al., 2023; Lou et al., 2024).

Another key signaling pathway: the WNT/β-catenin signaling pathway, critical for regulating cell proliferation, differentiation, and fate determination, is frequently dysregulated in carcinogenesis (Yen et al., 2019). Li et al. demonstrated that curcumin could exert inhibitory effects on hepatocellular carcinoma cancer via modulation of the WNT/β-catenin signaling pathway (Li et al., 2023). Dou et al found that curcumin inhibited cell proliferation by suppressing the Wnt/β-catenin pathway (Dou et al., 2017). Our network pharmacology analysis identified the Wnt/β-catenin signaling pathway as a key pathway in curcumin’s therapeutic action. We observed that curcumin promotes GSK3β phosphorylation, downregulates β-catenin levels, and thereby regulates the Wnt/β-catenin signaling pathway. Notably, while previous studies did not clarify the specific mechanism underlying WNT/β-catenin signaling pathway modulation in this context, we identified Ser-9 phosphorylation as the critical mediator.

Combining curcumin with oxaliplatin demonstrates superior efficacy compared to monotherapy, providing a rationale for evaluating this regimen in CRC patients. Curcumin has been demonstrated to synergistically potentiate the antitumor effects of multiple chemotherapeutic agents, including dasatinib, erlotinib, and the FOLFOX regimen (Nautiyal et al., 2011; Yamauchi et al., 2014). In our study, both curcumin and oxaliplatin monotherapies significantly inhibited the growth of CRC. Notably, the combination of curcumin and oxaliplatin exerted a synergistic growth-inhibitory effect, resulting in a greater reduction in tumor volume compared with the respective single-agent treatments. These findings suggest a potential synergistic antitumor mechanism. Consistent with these results, Guo et al. previously reported comparable observations, which further support the therapeutic potential of curcumin for clinical translation in CRC (Guo et al., 2015).

This study also has several limitations. First, although network pharmacology has emerged as a valuable tool in researching natural compounds, the incompleteness of existing public databases may result in insufficient analytical data. Second, while curcumin exerts a synergistic antitumor effect with oxaliplatin, the underlying mechanism remains to be elucidated. Last, it is imperative to conduct relevant clinical studies, including investigations into the combined application with oxaliplatin, to promote its clinical translation.

In this study, we demonstrated that curcumin regulate ferroptosis and Wnt/β-catenin signaling pathway in the treatment of CRC. It provides a more robust theoretical foundation for the clinical application and treatment of curcumin.

## Data Availability

The original contributions presented in the study are publicly available. This data can be found here: 10.6084/m9.figshare.30999640. Further inquiries can be directed to the corresponding author(s).

## Supplementary material

Supplementary Figure S1 The IC50 of curcumin in HCT116 and DLD1 cells.

Supplementary Figure S2 Utilization of the MCODE plugin based on 46 potential targets.

Supplementary Figure S3 Correlation between hub genes and immune cells based on GSE146771 Smart-seq2 dataset.

Supplementary Figure S4 Characteristics of prognostic genes. **(A)** Distribution map of patients in high and low risk groups based on risk score. **(B)** Survival status map of patients in high and low risk groups. **(C)** Heatmap of DR-DEGs associated with prognostic features of CRC. **(D)** Time-dependent ROC curve: 1 year, 3 years, and 5 years.

Supplementary Figure S5 Curcumin treatment significantly promotes the release of ROS in HCT116 cells and DLD1 cells, and this effect was remarkably reversed upon the addition of Fer-1.

Supplementary Figure S6 Both erastin and curcumin treatment significantly downregulated the expression of GPX4 and SLC7A11in HCT116 cells and DLD1 cells, while this effect of curcumin was reversed upon the addition of Fer-1.

Supplementary Figure S7 Curcumin treatment significantly downregulates the expression levels of SIRT1, SERPINE1, MMP3, WNT5A in HCT116 cells and DLD1 cells.

Supplementary Figure S8 Representative captures of immunocytochemistry indicated SLC7A11 or GPX4 expression in tumour tissues. ****P < 0.0001.
