# Supplementary figures and images for: Integrated network pharmacology and experimental verification to reveal the mechanisms of curcumin in the treatment of colorectal cancer
Source: Front Pharmacol. 2026 Jan 21;16:1703562. doi: 10.3389/fphar.2025.1703562 (PMC12868254; doi:10.3389/fphar.2025.1703562)

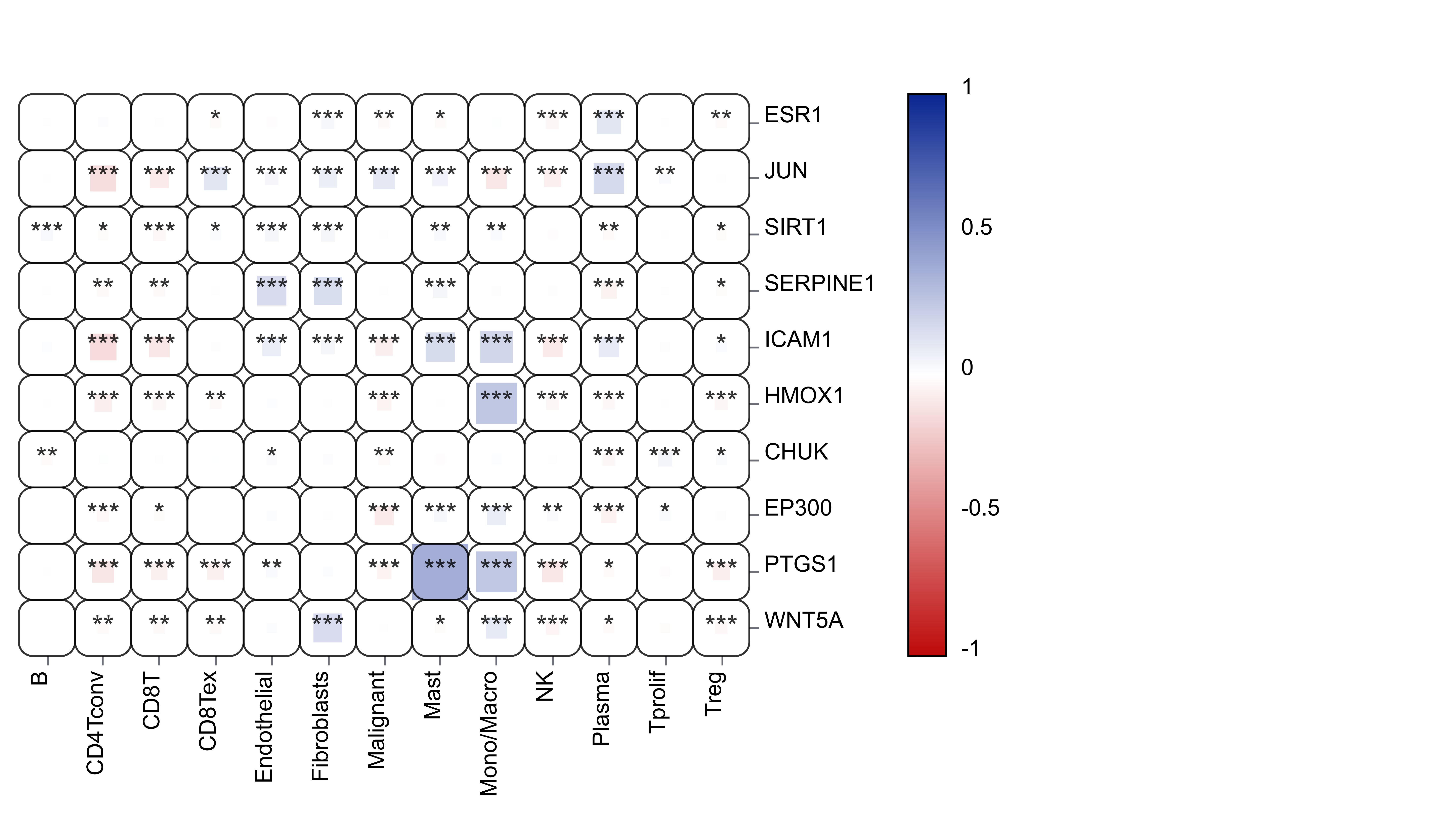

Supplement: Supplementary file 1 [file Image3.tiff]

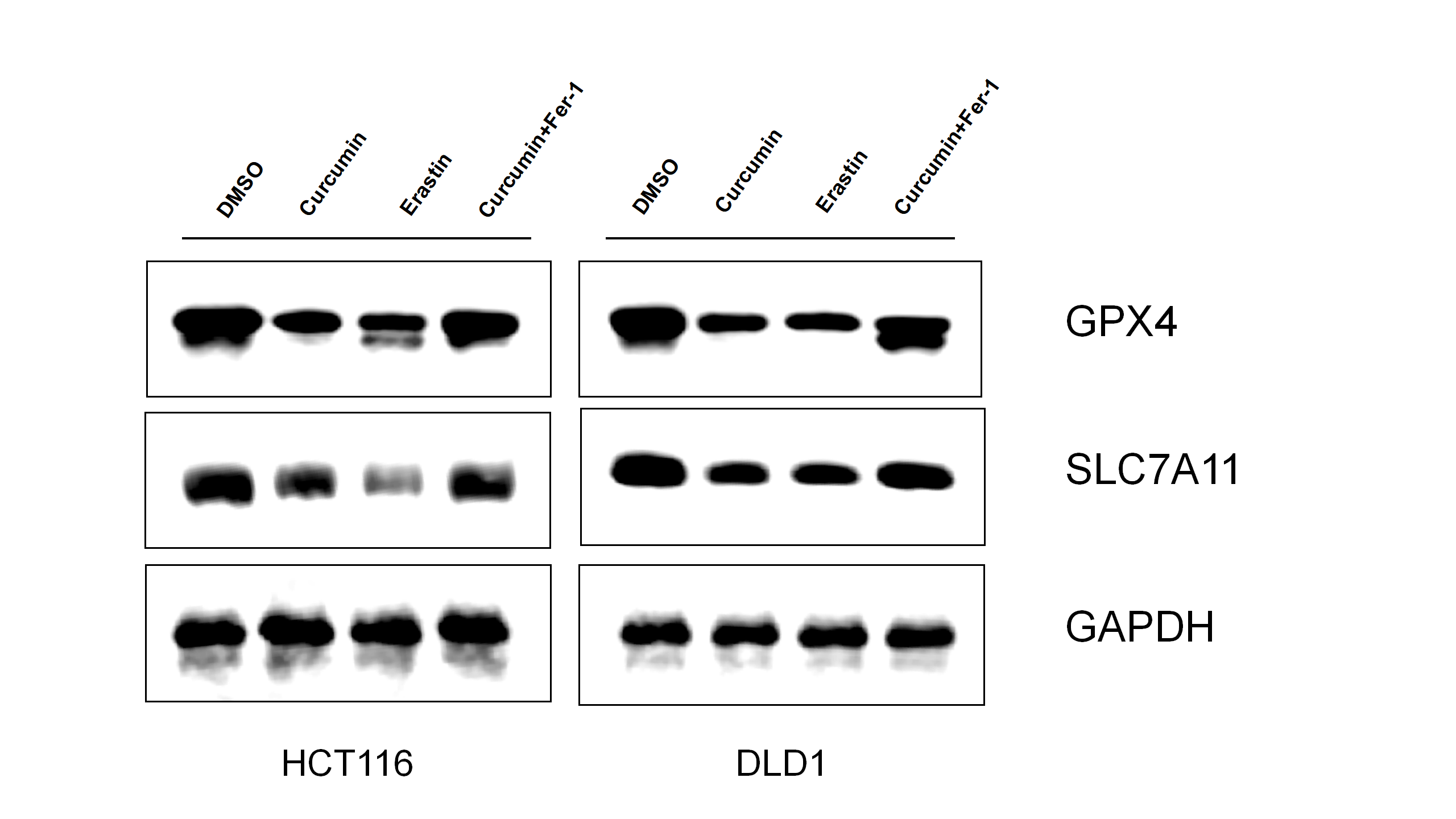

Supplement: Supplementary file 2 [file Image6.tif]

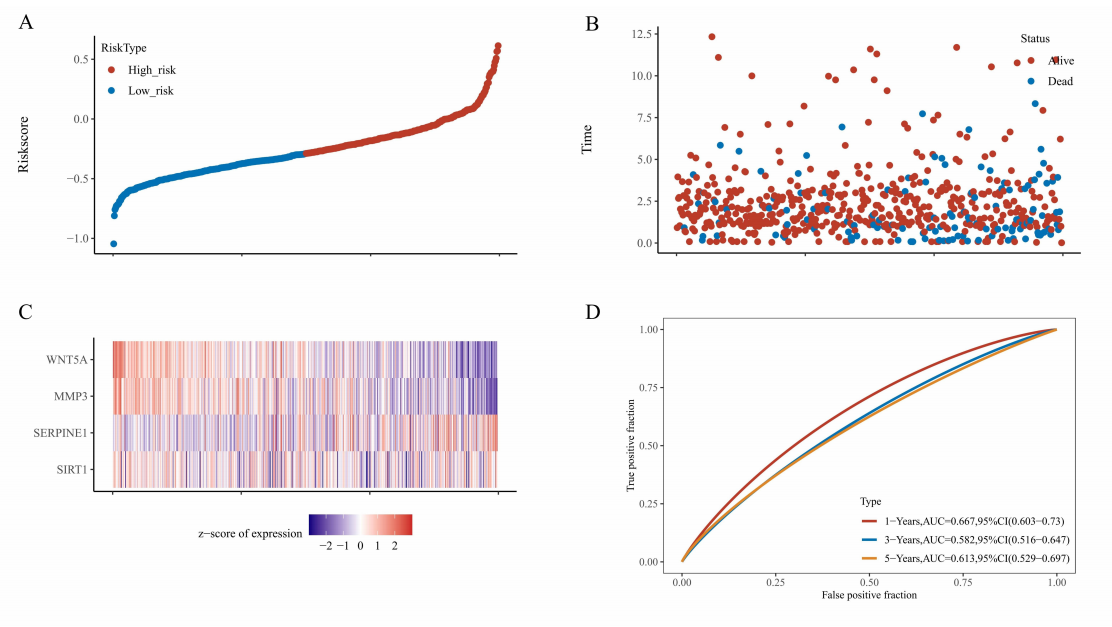

Supplement: Supplementary file 4 [file Image4.tif]

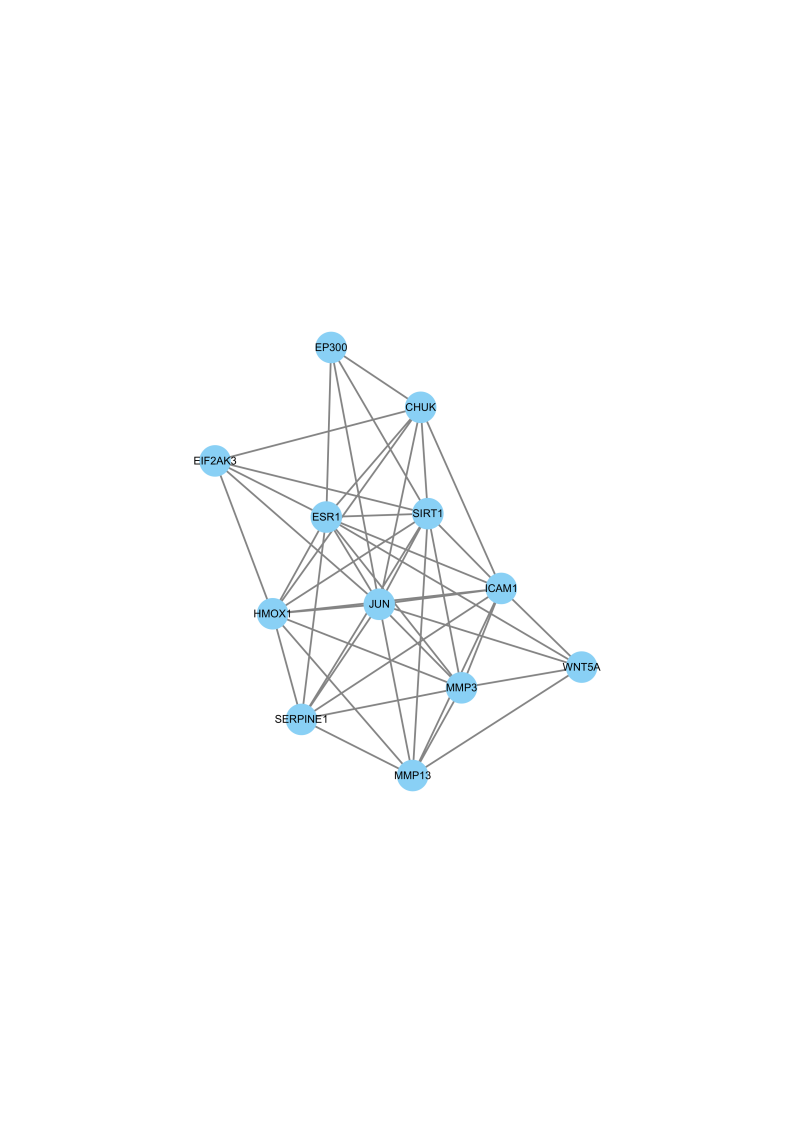

Supplement: Supplementary file 5 [file Image2.tif]

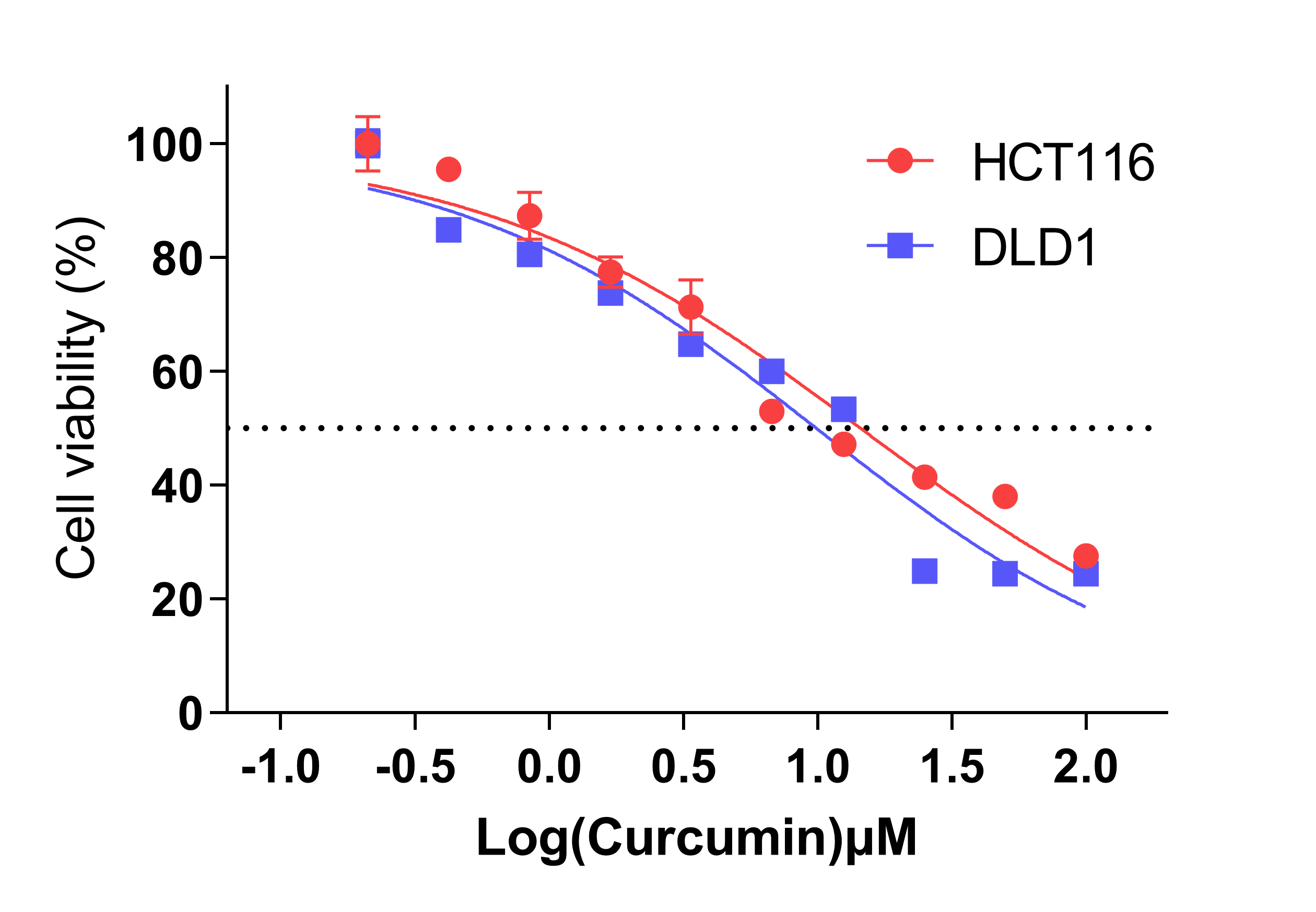

Supplement: Supplementary file 6 [file Image1.tif]

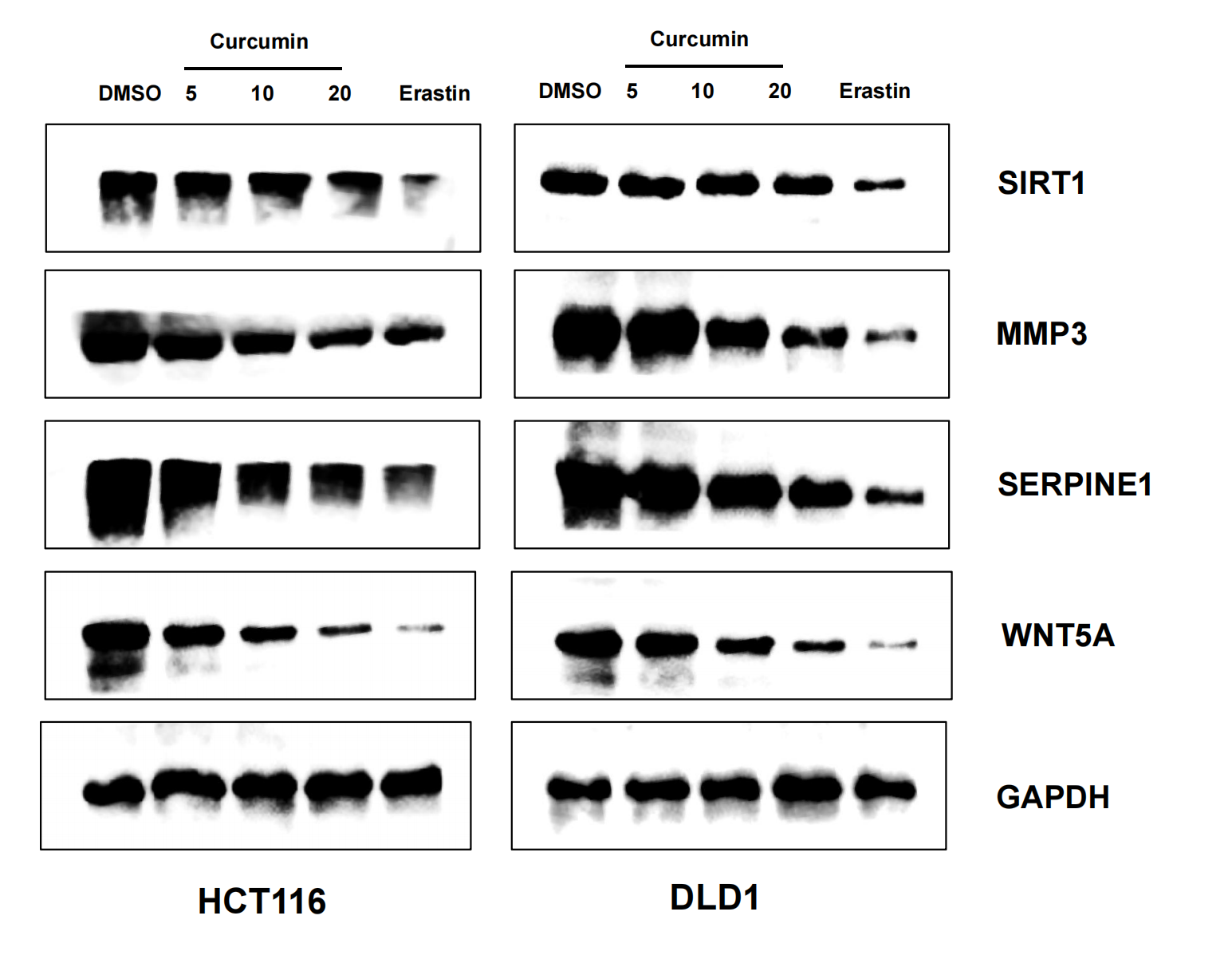

Supplement: Supplementary file 7 [file Image7.tif]

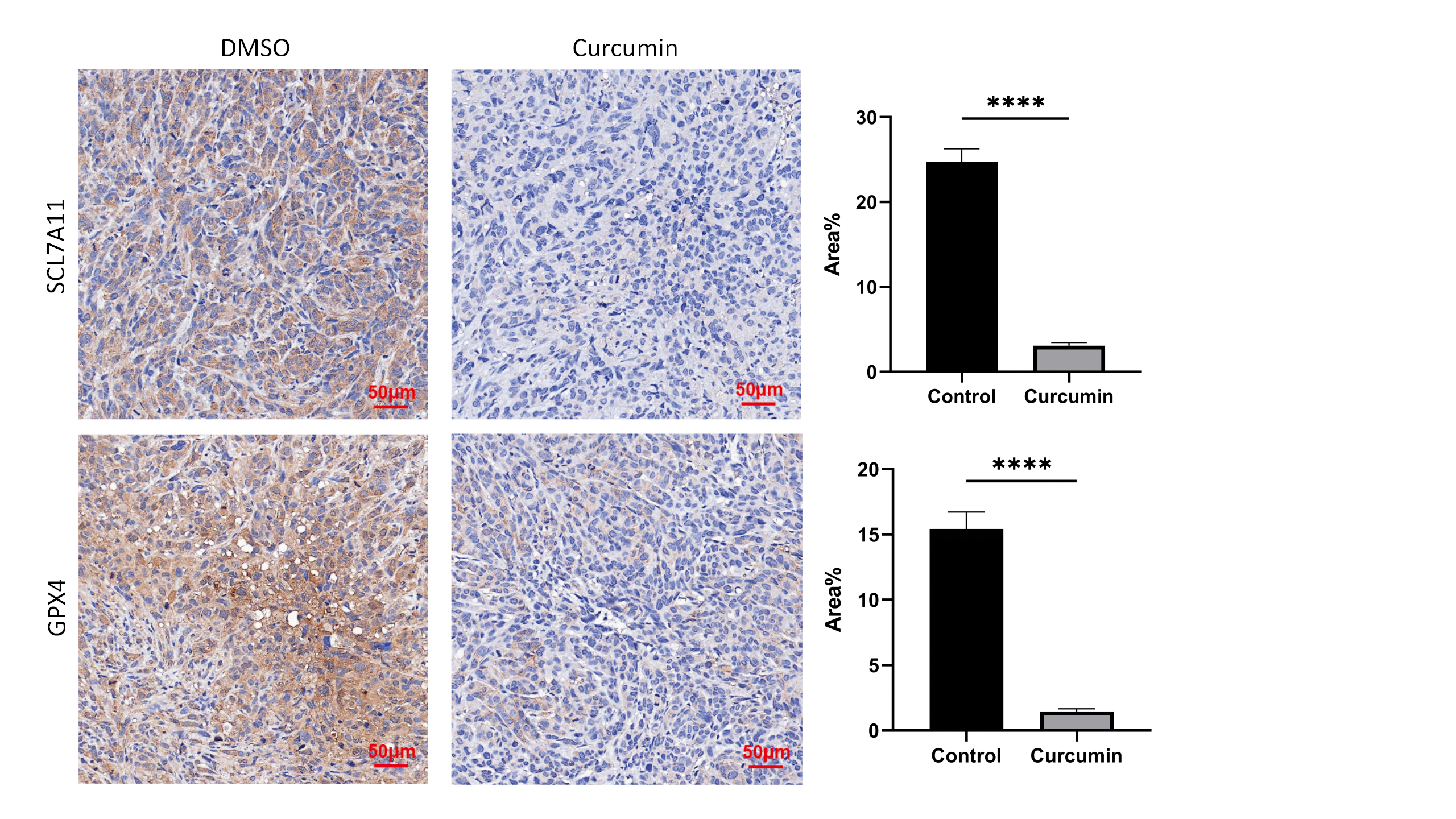

Supplement: Supplementary file 9 [file Image8.tif]

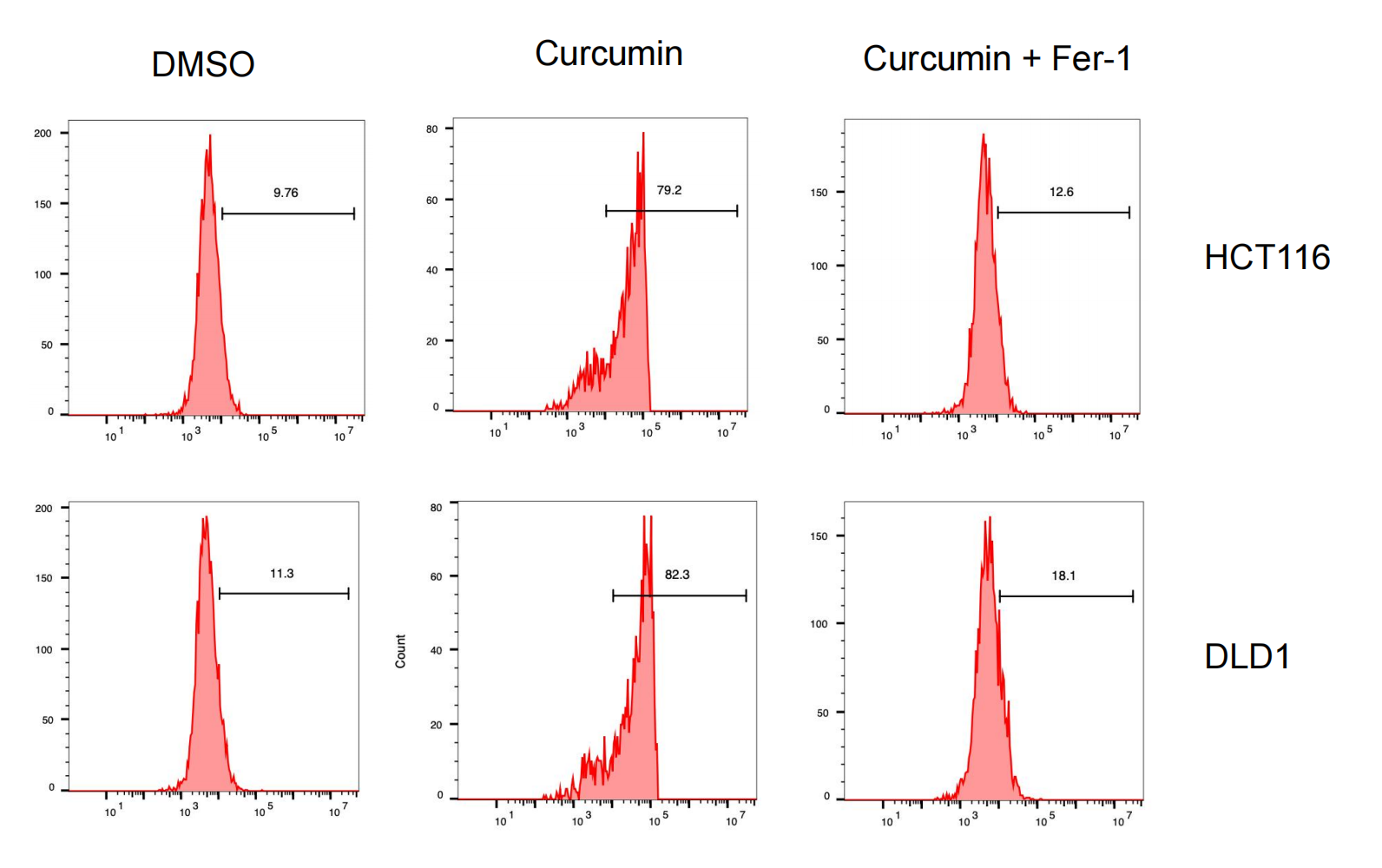

Supplement: Supplementary file 10 [file Image5.tif]
