# Supplementary material for: Integrated network pharmacology and experimental verification to reveal the mechanisms of curcumin in the treatment of colorectal cancer
Source: Front Pharmacol. 2026 Jan 21;16:1703562. doi: 10.3389/fphar.2025.1703562 (PMC12868254; doi:10.3389/fphar.2025.1703562)
Supplement: Supplementary file 3 [file Table1.docx]

| ACACB | CA12 | EP300 | MMP13 | SPHK1 | SHBG | DAPK1 | WNT5A |
| --- | --- | --- | --- | --- | --- | --- | --- |
| ADAM17 | CA2 | F2 | NAMPT | TOP1 | BST1 | PIM1 | GCLM |
| ADRA2A | CHRM1 | F3 | PRKCQ | TYMS | CLK1 | BTK | TF |
| ALOX12 | DHFR | HMOX1 | PTGS1 | ICAM1 | MMP3 | SIRT5 | SLC7A11 |
| ALOX15 | DUT | HTR1D | SCNN1A | CHUK | EPHA2 | SIRT1 |  |
| AURKB | EIF2AK3 | JUN | SERPINE1 | ESR1 | RAB5A | SOST |  |

**Supplement Table 1** Potential targets
