# Supplementary material for: Integrated network pharmacology and experimental verification to reveal the mechanisms of curcumin in the treatment of colorectal cancer
Source: Front Pharmacol. 2026 Jan 21;16:1703562. doi: 10.3389/fphar.2025.1703562 (PMC12868254; doi:10.3389/fphar.2025.1703562)
Supplement: Supplementary file 8 [file Table2.docx]

|  |  |  | CL(95%) | |
| --- | --- | --- | --- | --- |
|  | mean | SD | lower | upper |
| ESR1 | 0.008893302 | 0.064706839 | 0.007653602 | 0.010133003 |
| JUN | 0.365445164 | 0.329606875 | 0.359130319 | 0.37176001 |
| SIRT1 | 0.050028006 | 0.144054105 | 0.047268114 | 0.052787898 |
| SERPINE1 | 0.030409176 | 0.112390095 | 0.028255925 | 0.032562426 |
| ICAM1 | 0.180987303 | 0.244125288 | 0.176310175 | 0.185664431 |
| HMOX1 | 0.054002613 | 0.149522221 | 0.051137958 | 0.056867267 |
| CHUK | 0.046309936 | 0.146183453 | 0.043509248 | 0.049110623 |
| EP300 | 0.195388955 | 0.277044501 | 0.190081137 | 0.200696772 |
| PTGS1 | 0.025977573 | 0.095892415 | 0.024140397 | 0.027814748 |
| WNT5A | 0.004316596 | 0.042918755 | 0.003494327 | 0.005138864 |

**Supplement Table 2** The proportion of expression of core genes in GSE146771 Smart-seq2 dataset
